# Numerical investigation of a typhoid disease model in fuzzy environment

**DOI:** 10.1038/s41598-023-48405-w

**Published:** 2023-12-11

**Authors:** Fazal Dayan, Nauman Ahmed, Ali Hasan Ali, Muhammad Rafiq, Ali Raza

**Affiliations:** 1 Department of Mathematics, School of Science, University of Management and Technology, Lahore, Pakistan; 2 Department of Mathematics and Statistics, The University of Lahore, Lahore, Pakistan; 3 Department of Computer Science and Mathematics, Lebanese American University, Beirut, Lebanon; 4 Department of Mathematics, Near East University, Mathematics Research Center, Near East Boulevard, 99138 Nicosia/Mersin 10, Turkey; 5 Department of Mathematics, College of Education for Pure Sciences, University of Basrah, Basrah, 61001 Iraq; 6 Institute of Mathematics, University of Debrecen, Pf. 400, Debrecen, 4002 Hungary; 7 Department of Mathematics, Faculty of Science & Technology, University of Central Punjab, Lahore, Pakistan; 8 Department of Physical Sciences, The University of Chenab, Gujrat, Pakistan

**Keywords:** Biological models, Infectious diseases, Applied mathematics

## Abstract

Salmonella Typhi, a bacteria, is responsible for typhoid fever, a potentially dangerous infection. Typhoid fever affects a large number of people each year, estimated to be between 11 and 20 million, resulting in a high mortality rate of 128,000 to 161,000 deaths. This research investigates a robust numerical analytic strategy for typhoid fever that takes infection protection into consideration and incorporates fuzzy parameters. The use of fuzzy parameters acknowledges the variation in parameter values among individuals in the population, which leads to uncertainties. Because of their diverse histories, different age groups within this community may exhibit distinct customs, habits, and levels of resistance. Fuzzy theory appears as the most appropriate instrument for dealing with these uncertainty. With this in mind, a model of typhoid fever featuring fuzzy parameters is thoroughly examined. Two numerical techniques are developed within a fuzzy framework to address this model. We employ the non-standard finite difference (NSFD) scheme, which ensures the preservation of essential properties like dynamic consistency and positivity. Additionally, we conduct numerical simulations to illustrate the practical applicability of the developed technique. In contrast to many classical methods commonly found in the literature, the proposed approach exhibits unconditional convergence, solidifying its status as a dependable tool for investigating the dynamics of typhoid disease.

## Introduction

Typhoid is a result of typhus, a condition with identical symptoms. This endemic illness is brought on by the extremely pathogenic bacteria Salmonella typhi. This bacterium was disseminated by polluted water and other carriers. Typhoid is characterized by a persistent fever, a very poor appetite, vomiting, a very bad headache, and exhaustion. The incubation period for typhoid is 7 to 14 days. The patient’s intestine, which is where the germ naturally dwells, serves as its home. There is an increase in the number of mononuclear phagocytic cells in the blood. The patient’s blood culture is a key factor in determining how to treat typhoid. Chloramphenicol is ingested if the strain is amoxicillin sensitive. The oral dose of ciprofloxacin or norfloxacin is used to eradicate the issue in the asymptomatic carrier. Due to multi-drug resistance bacteria, antibiotic treatment has grown more challenging globally. In many nations, eliminating the disease will only be possible with the provision of clean, safe, and sanitary living circumstances, wholesome food, and the aforementioned medical services. These actions may lessen or eradicate the condition, though it is difficult to reach this goal. Following the implementation of health education initiatives that alter behavior toward illness prevention and treatment, the public can be made more aware. Every year, typhoid affects millions of people throughout the world. Typhoid is currently treated with oral and injectable vaccines, however, these two are insufficient to eradicate the illness. The length of the sickness can be shortened if a drug-resistant strain is used to treat the infected person^1,2^.

The dynamics of infectious diseases have been studied and explained using a variety of mathematical models^3^. To investigate the spread of infection at various endemicity levels, Cvjetanovic et al. built a mathematical model for typhoid disease. The model was used to predict the likely impact of typhoid fever prevention strategies, such as mass vaccination campaigns and sanitation programs, on a chosen population in terms of illness prevention as well as in terms of relative costs and benefits^4^. In order to examine the dynamics of typhoid fever sickness while including infection resistance, Nthiiri et al. developed a mathematical model. Using the next-generation matrix technique, the model’s stable states are identified and the reproduction number is calculated. The model’s stability is analyzed to identify the factors that contribute to the disease’s spread within a specific community^5^. The dynamics of the typhoid fever model were investigated, as well as the existence and uniqueness of the solution, by Peter et al. For the model, stability analysis is also carried out^6^. Bakach et al. reviewed some mathematical models of typhoid^7^. Karunditu et al. formulated a mathematical model of typhoid fever incorporating unprotected humans. The local and global stability of equilibrium points is also studied^8^. A mathematical model for the transmission of typhoid was developed by Nyaberi and Musaili, and it examines the effects of treatment on the dynamics of the illness^9^. Birger et al. studied mathematical models of typhoid transmission by considering FQNS and multidrug resistance separately. The effect of vaccination was predicted on the basis of forecasts of vaccine coverage^10^. A mathematical model for typhoid fever spread in a population is formulated. The equilibrium points of the model and their stabilities are investigated^11^. By utilizing several optimal control strategies, Wameko et al. established a mathematical model to look into the dynamics of typhoid disease. Typhoid disinformation among the population is reduced when the three control techniques are quickly implemented, as demonstrated^12^.

Due to population-wide variations in susceptibility, exposure, infectivity, and recovery, the parameters employed in epidemic models are imprecise. If different age groups, population groups, and resistance patterns are taken into account, differences may result. To take into consideration these varying degrees of persons, more realistic models are required. Mishra et al. claim that due to the high level of uncertainty, epidemic systems, particularly those involving infectious diseases, require a new approach^13^. Fuzzy sets and fuzzy logic have been extensively utilized to tackle real-world problems across diverse domains, encompassing medicine, engineering, economics, and numerous other fields where human decision-making plays a pivotal role in assessment and logical reasoning^14–18^, just to mention a few. Moreover, scholars have harnessed this theoretical framework in epidemiology as well. Incorporating fuzzy theory and treating the transmission coefficient as a fuzzy set, Barros et al. suggested a SI model^19^. Fuzzy logic was used by Ortega et al. to predict issues with infectious disease epidemiology. A rabies model in dogs with incomplete vaccinations was discussed^20^.

Mondal et al. developed an SIS model for investigating the plague using the fuzzy set theory^21^. Das and Pal developed a SIR model and studied it mathematically and numerically^22^. Sadhukhan et al. conducted research on harvesting optimization in a food chain model in a fuzzy environment^23^. To capture the dynamics of coronavirus illness, Li et al. developed a fuzzy SEIR model supplemented by confidence index theory^24^. Abdy et al. presented an SIR model that incorporated fuzzy parameters to depict the dynamics of COVID-19^25^. Furthermore, Allehiany et al. explored a fuzzy SIR model employing Euler, RK-4, and NSFD methods^26^. The NSFD approach, first described by Micken^27^, has been used by a number of researchers for solving systems of differential equations^28–30^, to name a few. Adak and Jana investigated an SIS model involving treatment control with the utilization of fuzzy numbers^31^.

The existing mathematical models of typhoid are insufficient for the advancement of fuzzy numerical and mathematical procedures. We investigated a typhoid model with fuzzy parameters with this in mind. We can cope with the difficulties of uncertainty quantification in mathematical disease modeling by using fuzzy theory. As a result, the introduction of fuzzy parameters aids in our ability to comprehend the dynamics of typhoid transmission. Even the biological factors employed in mathematical models are not always constant because each community changes as the environment change. The majority of the issues linked to the rise in the earth’s average temperature are caused by global warming. The rate at which the virus spreads throughout the population is also impacted by temperature changes. Fuzzy mathematical models are more insightful than crisp models in this regard. The creation, use, and analysis of first order explicit numerical techniques in fuzzy non-standard finite difference situations are novel aspects of the current work.

The rest of this study is organized as follows: The formulation of the fuzzy model is discussed in the following section. Within the same section, we discuss equilibrium analysis, stability analysis, and the fuzzy basic reproduction number. Following that, in the numerical modeling section, we elaborate on the creation of the forward Euler scheme and NSFD schemes for the examined model. This section also includes an assessment of the NSFD scheme’s stability and consistency. The next part displays numerical simulations involving the developed techniques. Finally, in the final section, we summarize concluding observations and outline future research directions.

## Typhoid fever model with fuzzy parameters

We examined the mathematical model previously discussed by Arif et al.^2^

1 $$\begin{aligned} {\frac{dT_1}{dt}}& = {\alpha \mu -(\gamma +\mu )T_1 }, \end{aligned}$$

2 $$\begin{aligned} {\frac{dT_2}{dt}}& = {(1-\alpha )\mu +\gamma T_1-\theta T_2 T_3- \mu T_2},\end{aligned}$$

3 $$\begin{aligned} {\frac{dT_3}{dt}}& = {\theta T_2 T_3-(\delta +\beta +\mu )T_3},\end{aligned}$$

4 $$\begin{aligned} {\frac{dT_4}{dt}}& = {\beta T_3-\mu T_4}.\end{aligned}$$

**Figure 1:**
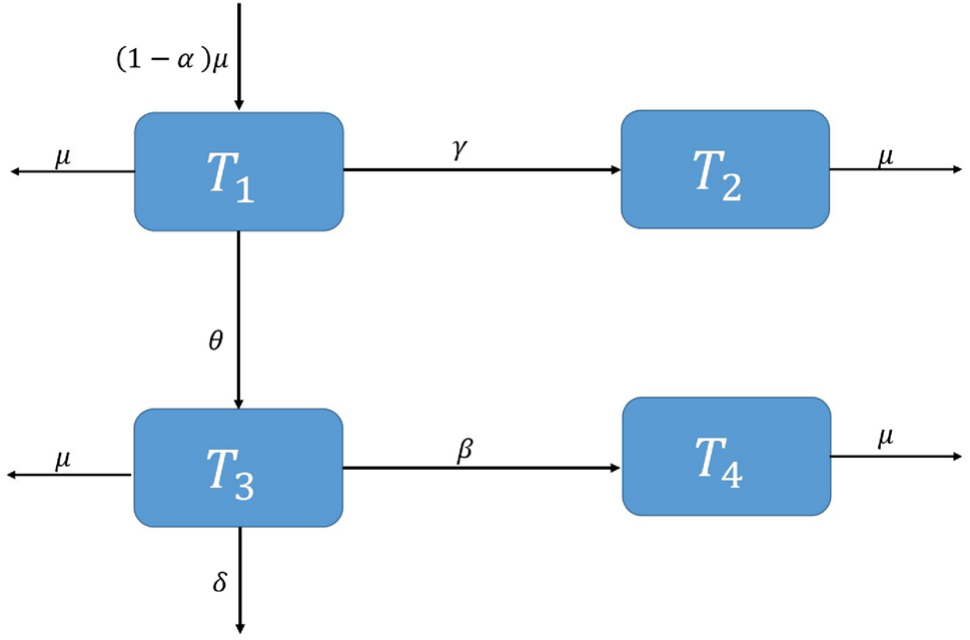
Flowchart of the model.

Here, $T_1$ represents the proportion of humans who are protected, $T_2$ represents the proportion of humans who are susceptible, $T_3$ signifies the fraction of humans who are currently infected, and $T_4$ indicates the fraction of humans receiving treatment. The rate at which humans receiving treatment transitions from the fraction of infected humans is denoted as $\beta$. The variable $\alpha$ represents the rate at which individuals enroll in the protected human compartment against typhoid, while $(1-\alpha )$ denotes the rate of individuals who remain susceptible to the virus, $\delta$ signifies the rate at which individuals experience a transient phase due to typhoid fever, and $\theta$ reflects the per capita rate at which susceptible individuals contract typhoid fever infection. Lastly, $\mu$ represents the natural rate of human death and birth. The depiction of the communication dynamics in the typhoid fever model is presented in Fig. 1.

The fuzzy representation of the model mentioned above can be expressed as follows:

5 $$\begin{aligned} {\frac{dT_1}{dt}}& = {\alpha \mu -(\gamma +\mu )T_1 },\end{aligned}$$

6 $$\begin{aligned} {\frac{dT_2}{dt}}& = {(1-\alpha )\mu +\gamma T_1-\theta (\Omega ) T_2 T_3- \mu T_2},\end{aligned}$$

7 $$\begin{aligned} {\frac{dT_3}{dt}}& = {\theta (\Omega ) T_2 T_3-(\delta +\beta (\Omega )+\mu )T_3},\end{aligned}$$

8 $$\begin{aligned} {\frac{dT_4}{dt}}& = {\beta (\Omega ) T_3-\mu T_4}.\end{aligned}$$

We assume that susceptible humans contract typhoid fever infection at a per capita rate denoted by $\theta (\Omega )$ and the fraction of treated humans stemming from infected individuals, $\beta (\Omega )$, are represented as fuzzy numbers, contingent upon the individual’s virus load. The variable $\theta (\Omega )$ can be defined as^19^

9 $$\begin{aligned} \theta (\Omega )= {\left\{ \begin{array}{lll} 0, & \quad \Omega < \Omega _{min}, \\ \frac{\Omega -\Omega _{min}} {\Omega _M-\Omega _{min}}, & \quad \Omega _{min} \le \Omega \le \Omega _M, \\ 1, & \quad \Omega _M < \Omega , \end{array}\right. } \end{aligned}$$

The $\theta (\Omega )$ reaches its peak when $\Omega$ is at its maximum value, and it becomes insignificant when $\Omega$ is at its minimum. $\Omega _{min}$ represents the minimum virus load required for disease transmission, and disease transmission is at its highest when $\Omega$ equals $\Omega _M$, reaching a value of 1. Similarly, $\beta (\Omega )$ can be defined as^32^

10 $$\begin{aligned} \beta (\Omega )= {\left\{ \begin{array}{ll} \frac{\beta _0-1}{\Omega _M} \Omega +1, \hspace{1cm} 0\le \Omega \le \Omega _M, \end{array}\right. } \end{aligned}$$

where $\beta _0>0$ is the minimum treatment rate.

### Equilibrium analysis

The analyzed model possesses a disease-free equilibrium point (DFE) and two endemic equilibrium points (EE).

**Case 1.**: If $\Omega <\Omega _{min}$ and $\beta (\Omega )>0$ then $\theta (\Omega )=0$ and we obtain,
  $$\begin{aligned} T^0(T_1^0, T_2^0, T_3^0, T_4^0)=\bigg (\frac{\alpha \mu }{\gamma +\mu }, \frac{(\gamma +\mu )(1-\alpha )+\gamma \alpha }{\gamma +\mu }, 0, 0\bigg ). \end{aligned}$$
  In this scenario, the human population remains free from typhoid, and this state is referred to as the DFE. From a biological perspective, typhoid disease is considered eradicated when the disease concentration within the population falls below the minimum threshold required for its sustained existence.
**Case 2.**: If $\Omega _{min}\le \Omega \le \Omega _M$ and $\beta (\Omega )>0$ then $\theta (\Omega )=\frac{\Omega -\Omega _{min}}{\Omega _M-\Omega _{min}}$ and we obtain,
  $$\begin{aligned} T^*=(T_1^*, T_2^*, T_3^*, T_4^*), \end{aligned}$$
  where
  $$\begin{aligned} T_1^*& = \frac{\alpha \mu }{\gamma +\mu },\\ T_2^*& = \frac{\delta +\beta (\Omega )+\mu }{\theta (\Omega )},\\ T_3^*& = \frac{(\gamma +\mu )(1-\alpha )\mu \theta (\Omega )+\gamma \alpha \mu \theta (\Omega )-\mu (\delta +\beta (\Omega )+\mu )(\gamma +\mu )}{\theta (\Omega )(\gamma +\mu )(\delta +\beta (\Omega )+\mu )}, \end{aligned}$$
  and
  $$\begin{aligned} T_4^*=\frac{\beta (\Omega )\bigg ((\gamma +\mu )(1-\alpha )\mu \theta (\Omega )+\gamma \alpha \mu \theta (\Omega )-\mu (\delta +\beta (\Omega )+\mu )(\gamma +\mu )\bigg )}{\theta (\Omega )(\gamma +\mu )(\delta +\beta (\Omega )+\mu )}. \end{aligned}$$
**Case 3.**: If $\Omega _M<\Omega$ and $\beta (\Omega )>0$ then $\theta (\Omega )=1$, and we obtain
  $$\begin{aligned} T^{**}=(T_1^{**}, T_2^{**}, T_3^{**}, T_4^{**}), \end{aligned}$$
  where
  $$\begin{aligned} T_1^{**}& = \frac{\alpha \mu }{\gamma +\mu },\\ T_2^{**}& = \frac{\delta +\beta (\Omega )+\mu }{\theta (\Omega )},\\ T_3^{**}& = \frac{(\gamma +\mu )(1-\alpha )\mu \theta (\Omega )+\gamma \alpha \mu \theta (\Omega )-\mu (\delta +\beta (\Omega )+\mu )(\gamma +\mu )}{\theta (\Omega )(\gamma +\mu )(\delta +\beta (\Omega )+\mu )}, \end{aligned}$$
  and
  $$\begin{aligned} T_4^{**}=\frac{\beta (\Omega )\bigg ((\gamma +\mu )(1-\alpha )\mu \theta (\Omega )+\gamma \alpha \mu \theta (\Omega )-\mu (\delta +\beta (\Omega )+\mu )(\gamma +\mu )\bigg )}{\theta (\Omega )(\gamma +\mu )(\delta +\beta (\Omega )+\mu )}. \end{aligned}$$

The points $$T^*$$ and $$T^{**}$$ represent situations where the prevalence of typhoid disease exceeds the minimum threshold necessary for its propagation, resulting in the persistence of typhoid within the human population.

### Stability analysis

To check the stability, let us assume the following system:

11 $$\begin{aligned} A_1& = {\alpha \mu -(\gamma +\mu )T_1 }, \end{aligned}$$

12 $$\begin{aligned} A_2& = {(1-\alpha )\mu +\gamma T_1-\theta (\Omega ) T_2 T_3- \mu T_2}, \end{aligned}$$

13 $$\begin{aligned} A_3& = {\theta (\Omega ) T_2 T_3-(\delta +\beta (\Omega )+\mu )T_3}, \end{aligned}$$

and

14 $$\begin{aligned} A_4={\beta (\Omega ) T_3-\mu T_4}. \end{aligned}$$

The Jacobian of the system (11)–(14) can be represented as:

$$\begin{aligned} J& = \left[ \begin{array}{cccc} \frac{\partial A_1}{\partial T_1} &{} \frac{\partial A_1}{\partial T_2} &{} \frac{\partial A_1}{\partial T_3} &{} \frac{\partial A_1}{\partial T_4} \\ \frac{\partial A_2}{\partial T_1} &{} \frac{\partial A_2}{\partial T_2} &{} \frac{\partial A_2}{\partial T_3} &{} \frac{\partial A_2}{\partial T_4} \\ \frac{\partial A_3}{\partial T_1} &{} \frac{\partial A_3}{\partial T_2} &{} \frac{\partial A_3}{\partial T_3} &{} \frac{\partial A_3}{\partial T_4} \\ \frac{\partial A_4}{\partial T_1} &{} \frac{\partial A_4}{\partial T_2} &{} \frac{\partial A_4}{\partial T_3} &{} \frac{\partial C_4}{\partial T_4} \\ \end{array} \right] , \\ J& = \left[ \begin{array}{cccc} -(\gamma +\mu ) &{} 0 &{}0 &{}0 \\ \gamma &{} -(\theta (\Omega ) T_3+\mu ) &{}-\theta (\Omega )T_2 &{} 0 \\ 0&{} \theta (\Omega ) T_3 &{} \theta (\Omega )T_2-(\delta +\beta (\Omega )+\mu ) &{} 0 \\ 0 &{}0 &{}\beta (\Omega ) &{} -\mu \\ \end{array} \right] . \end{aligned}$$

The Jacobian at the DFE is

$$\begin{aligned}J=\left[ \begin{array}{cccc} -(\gamma +\mu ) &{} 0 &{}0 &{}0 \\ \gamma &{} -\mu &{}0 &{} 0 \\ 0&{} \theta (\Omega ) T_3 &{} -(\delta +\beta (\Omega )+\mu ) &{} 0 \\ 0 &{}0 &{}\beta (\Omega ) &{} -\mu \\ \end{array} \right] . \end{aligned}$$

The local asymptotic stability of the steady-state is confirmed when the absolute eigenvalues of the Jacobian matrix mentioned earlier having negative real parts. Analyzing the Jacobian matrix, we find that the eigenvalues are as follows: $\lambda _1= -(\gamma +\mu )$, $\lambda _2 = \lambda _4= - \mu$, and $\lambda _3 =- (\delta +\beta (\Omega )+\mu )$. The fact that all of these eigenvalues having negative real parts validates the desired result.

### Fuzzy basic reproductive number $R_t^f$

Using the next-generation matrix technique, we calculated the reproductive number, denoted as $R_t$.

15 $$\begin{aligned} R_t=\frac{\theta (\Omega )(\gamma +\mu -\alpha \mu )}{(\gamma +\mu )(\delta +\beta (\Omega )+\mu )}. \end{aligned}$$

Verma et al.^33^ examined different scenarios by manipulating the parameters and, in each case, determined the reproduction number to assess whether the virus spread was effectively managed.

Now, $R_t$, being a function of the typhoid disease, can be analyzed as follows^34^:

**Case 1.**: If $\Omega <\Omega _{min}$ and $\beta (\Omega )>0$ then $\theta (\Omega )=0$ and $R_t(\Omega )=0$.
**Case 2.**: If $\Omega _{min}\le \Omega \le \Omega _M$ and $\beta (\Omega )>0$ then $\theta (\Omega )=\frac{\Omega -\Omega _{min}}{\Omega _M-\Omega _{min}}$ and $R_t(\Omega )=\frac{\theta (\Omega )(\gamma +\mu -\alpha \mu )}{(\gamma +\mu )(\delta +\beta (\Omega )+\mu )}$.
**Case 3.**: If $\Omega _M<\Omega$ and $\beta (\Omega )>0$ then $\theta (\Omega )=1$ and $R_t(\Omega )=\frac{\gamma +\mu -\alpha \mu }{(\gamma +\mu )(\delta +\beta (\Omega )+\mu )}$. The disease-dependent function $R_t(\Omega )$ correlates positively with the disease parameter $\Omega$, and its definition includes a fuzzy variable. As a result, the expected value of $R_t(\Omega )$ is well defined, and its representation can be written as a triangular fuzzy number, as follows:
  16 $$\begin{aligned} R_t(\Omega )=\bigg (0, \frac{\theta (\Omega )(\gamma +\mu -\alpha \mu )}{(\gamma +\mu )(\delta +\beta (\Omega )+\mu )}, \frac{\gamma +\mu -\alpha \mu }{(\gamma +\mu )(\delta +\beta (\Omega )+\mu )}\bigg ). \end{aligned}$$
  Now, $R_t^f$ can be found as
  17 $$\begin{aligned} R_t^f=E[R_t (\Omega )], \end{aligned}$$
  and therefore,
  18 $$\begin{aligned} R_t^f=\frac{(\gamma +\mu -\alpha \mu )(2\theta (\Omega )+1)}{4(\gamma +\mu (\delta +\beta (\Omega )+\mu )}. \end{aligned}$$

## Numerical modeling

### Forward Euler scheme

The Forward Euler scheme is a well-known explicit first-order numerical approach for solving ordinary differential equations. It is computationally efficient and provides a rapid estimation of the behavior of solutions over time.

19 $$\begin{aligned} T_1^{n+1}& = T_1^n+h[\alpha \mu -(\gamma +\mu ) T_1^n ], \end{aligned}$$

20 $$\begin{aligned} T_2^{n+1}& = T_2^n+h[(1-\alpha )\mu +\gamma T_1^n-\theta (\Omega ) T_2^n T_3^n-\mu T_2^n ], \end{aligned}$$

21 $$\begin{aligned} T_3^{n+1}& = T_3^n+h[\theta (\Omega ) T_2^n T_3^n-(\delta +\beta (\Omega )+\mu ) T_3^n ], \end{aligned}$$

22 $$\begin{aligned} T_4^{n+1}& = T_4^n+h[\beta (\Omega )) T_3^n-\mu T_4^n ]. \end{aligned}$$

**Case 1.**: If $\Omega <\Omega _{min}$ and $\beta (\Omega )>0$ then $\theta (\Omega )=0$ and
  23 $$\begin{aligned} T_1^{n+1}& = T_1^n+h[\alpha \mu -(\gamma +\mu ) T_1^n ],\end{aligned}$$
  24 $$\begin{aligned} T_2^{n+1}& = T_2^n+h[(1-\alpha )\mu +\gamma T_1^n-\mu T_2^n ], \end{aligned}$$
  25 $$\begin{aligned} T_3^{n+1}& = T_3^n-h[(\delta +\beta (\Omega )-\mu ) T_3^n ], \end{aligned}$$
  26 $$\begin{aligned} T_4^{n+1}& = T_4^n+h[\beta (\Omega )) T_3^n-\mu T_4^n ].\end{aligned}$$
**Case 2.**: If $\Omega _{min}\le \Omega \le \Omega _M$ and $\beta (\Omega )>0$ then $\theta (\Omega )=\frac{\Omega -\Omega _{min}}{\Omega _M-\Omega _{min}}$ and the Euler scheme becomes
  27 $$\begin{aligned} T_1^{n+1}& = T_1^n+h[\alpha \mu -(\gamma +\mu ) T_1^n ],\end{aligned}$$
  28 $$\begin{aligned} T_2^{n+1}& = T_2^n+h[(1-\alpha )\mu +\gamma T_1^n-\theta (\Omega ) T_2^n T_3^n-\mu T_2^n ], \end{aligned}$$
  29 $$\begin{aligned} T_3^{n+1}& = T_3^n+h[\theta (\Omega ) T_2^n T_3^n-(\delta +\beta (\Omega )+\mu ) T_3^n ], \end{aligned}$$
  30 $$\begin{aligned} T_4^{n+1}& = T_4^n+h[\beta (\Omega )) T_3^n-\mu T_4^n ].\end{aligned}$$
**Case 3.**: If $\Omega _M<\Omega$ and $\beta (\Omega )>0$ then $\theta (\Omega )=1$ and
  31 $$\begin{aligned} T_1^{n+1}& = T_1^n+h[\alpha \mu -(\gamma +\mu ) T_1^n ],\end{aligned}$$
  32 $$\begin{aligned} T_2^{n+1}& = T_2^n+h[(1-\alpha )\mu +\gamma T_1^n- T_2^n T_3^n-\mu T_2^n ], \end{aligned}$$
  33 $$\begin{aligned} T_3^{n+1}& = T_3^n+h[ T_2^n T_3^n-(\delta +\beta (\Omega )+\mu ) T_3^n ], \end{aligned}$$
  34 $$\begin{aligned} T_4^{n+1}& = T_4^n+h[\beta (\Omega )) T_3^n-\mu T_4^n ].\end{aligned}$$

### Non-standard finite difference (NSFD) scheme

The NSFD scheme is a class of numerical methods for approximating solutions to differential equations. These approaches differ from traditional finite difference methods in their approach to discretizing the domain and approximating derivatives. It has the potential to improve accuracy in the solution of differential equations. The NSFD numerical model is formulated based on the NSFD theory introduced by Mickens^35^.

35 $$\begin{aligned} T_1^{n+1}& = \frac{T_1^n+h\alpha \mu }{1+h(\gamma +\mu )}, \end{aligned}$$

36 $$\begin{aligned} T_2^{n+1}& = \frac{T_2^n+h((1-\alpha )\mu +\gamma T_1^n)}{1+h(\theta (\Omega )T_3^n +\mu )}, \end{aligned}$$

37 $$\begin{aligned} T_3^{n+1}& = \frac{T_3^n+h\theta (\Omega ) T_2^n T_3^n}{1+h(\delta +\beta (\Omega )+\mu )}, \end{aligned}$$

38 $$\begin{aligned} T_4^{n+1}& = \frac{T_4^n+h\beta (\Omega ) T_3^n}{1+h\mu }. \end{aligned}$$

**Case 1.**: If $\Omega <\Omega _{min}$ and $\beta (\Omega )>0$ then $\theta (\Omega )=0$ and the NSFD scheme becomes
  39 $$\begin{aligned} T_1^{n+1}& = \frac{T_1^n+h\alpha \mu }{1+h(\gamma +\mu )},\end{aligned}$$
  40 $$\begin{aligned} T_2^{n+1}& = \frac{T_2^n+h(1-\alpha )\mu +\gamma T_1^n)}{1+h\mu },\end{aligned}$$
  41 $$\begin{aligned} T_3^{n+1}& = \frac{T_3^n}{1+h(\delta +\beta (\Omega )+\mu )}, \end{aligned}$$
  42 $$\begin{aligned} T_4^{n+1}& = \frac{T_4^n+h\beta (\Omega ) T_3^n}{1+h\mu }.\end{aligned}$$
**Case 2.**: If $\Omega _{min}\le \Omega \le \Omega _M$ and $\beta (\Omega )>0$ then $\theta (\Omega )=\frac{\Omega -\Omega _{min}}{\Omega _M-\Omega _{min}}$ and the NSFD Scheme becomes
  43 $$\begin{aligned} T_1^{n+1}& = \frac{T_1^n+h\alpha \mu }{1+h(\gamma +\mu )},\end{aligned}$$
  44 $$\begin{aligned} T_2^{n+1}& = \frac{T_2^n+h(1-\alpha )\mu +\gamma T_1^n)}{1+h(\theta (\Omega )T_3^n +\mu )},\end{aligned}$$
  45 $$\begin{aligned} T_3^{n+1}& = \frac{T_3^n+h\theta (\Omega ) T_2^n T_3^n}{1+h(\delta +\beta (\Omega )+\mu )}, \end{aligned}$$
  46 $$\begin{aligned} T_4^{n+1}& = \frac{T_4^n+h\beta (\Omega ) T_3^n}{1+h\mu }.\end{aligned}$$
**Case 3.**: If $\Omega _M<\Omega$ and $\beta (\Omega )>0$ then $\theta (\Omega )=1$ and the scheme becomes
  47 $$\begin{aligned} T_1^{n+1}& = \frac{T_1^n+h\alpha \mu }{1+h(\gamma +\mu )},\end{aligned}$$
  48 $$\begin{aligned} T_2^{n+1}& = \frac{T_2^n+h(1-\alpha )\mu +\gamma T_1^n)}{1+h(T_3^n +\mu )},\end{aligned}$$
  49 $$\begin{aligned} T_3^{n+1}& = \frac{T_3^n+h T_2^n T_3^n}{1+h(\delta +\beta (\Omega )+\mu )}, \end{aligned}$$
  50 $$\begin{aligned} T_4^{n+1}& = \frac{T_4^n+h\beta (\Omega ) T_3^n}{1+h\mu }.\end{aligned}$$

#### Consistency analysis

To check the consistency of the proposed scheme, we start by taking Eq. (35), and we have

51 $$\begin{aligned} T_1^{n+1}[1+h(\gamma +\mu )]=T_1^n+h\alpha \mu . \end{aligned}$$

Taking into account the Taylor’s series expansion for $T_1^{n+1}$,

52 $$\begin{aligned} T_1^{n+1}=T_1^n+h \frac{dT_1}{dt}+\frac{h^2}{2!} \frac{d^2T_1}{dt^2}+\frac{h^3}{3!} \frac{d^3T_1}{dt^3} +\dots . \end{aligned}$$

Substituting the value of $T_1^{n+1}$ in Eq. (51), we get

53 $$\begin{aligned} \bigg (T_1^n+h \frac{dT_1}{dt}+\frac{h^2}{2!} \frac{d^2T_1}{dt^2}+\frac{h^3}{3!} \frac{d^3T_1}{dt^3} +\dots \bigg )\big [1+h(\gamma +\mu )\big ]=T_1^n+h\alpha \mu . \end{aligned}$$

Taking $h\rightarrow 0$, we get

54 $$\begin{aligned} \frac{dT_1}{dt}+(\gamma +\mu )T_1^n=\alpha \mu , \end{aligned}$$

or

55 $$\begin{aligned} {\frac{dT_1}{dt}}={\alpha \mu -(\gamma +\mu )T_1 }. \end{aligned}$$

From Eq. (36), we have

56 $$\begin{aligned} T_2^{n+1} [1+h(\theta (\Omega )T_3^n +\mu )]=T_2^n+h((1-\alpha )\mu +\gamma T_1^n). \end{aligned}$$

Taking into account the Taylor’s series expansion for $T_2^{n+1}$,

57 $$\begin{aligned} T_2^{n+1}=T_2^n+h \frac{dT_2}{dt}+\frac{h^2}{2!} \frac{d^2T_2}{dt^2}+\frac{h^3}{3!} \frac{d^3T_2}{dt^3} + \dots . \end{aligned}$$

Substituting the value of $T_2^{n+1}$ in Eq. (56), we get

58 $$\begin{aligned} \bigg (T_2^n+h \frac{dT_2}{dt}+\frac{h^2}{2!} \frac{d^2T_2}{dt^2}+\frac{h^3}{3!} \frac{d^3T_2}{dt^3} +\dots \bigg ) \Big [1+h(\theta (\Omega )T_3^n +\mu )\Big ]=T_2^n+h((1-\alpha )\mu +\gamma T_1^n). \end{aligned}$$

Taking $h\rightarrow 0$, we get

59 $$\begin{aligned}{} & {} (\theta (\Omega )T_3^n+\mu )T_2^n+\frac{dT_2}{dt}=(1-\alpha )\mu +\gamma T_1^n, \end{aligned}$$

60 $$\begin{aligned}{} & {} \frac{dT_2}{dt}=(1-\alpha )\mu +\gamma T_1^n -(\theta (\Omega )T_3^n+\mu )T_2^n.\end{aligned}$$

From Eq. (37), we have

61 $$\begin{aligned} T_3^{n+1} (1+h(\delta +\beta (\Omega )+\mu )) =T_3^n+h\theta (\Omega ) T_2^n T_3^n. \end{aligned}$$

Similarly, taking into account the Taylor’s series expansion for $T_3^{n+1}$,

62 $$\begin{aligned} T_3^{n+1}=T_3^n+h \frac{dT_3}{dt}+\frac{h^2}{2!} \frac{d^2T_3}{dt^2}+\frac{h^3}{3!} \frac{d^3T_3}{dt^3} +\dots . \end{aligned}$$

Substituting the value of $T_3^{n+1}$ in Eq. (61), we get

63 $$\begin{aligned} \bigg (T_3^n+h \frac{dT_3}{dt}+\frac{h^2}{2!} \frac{d^2T_3}{dt^2}+\frac{h^3}{3!} \frac{d^3T_3}{dt^3} +\dots \bigg )(1+h(\delta +\beta (\Omega )+\mu )) =T_3^n+h\theta (\Omega ) T_2^n T_3^n. \end{aligned}$$

Simplifying Eq. (63) and taking $h\rightarrow 0$, we get

64 $$\begin{aligned} \frac{dT_3}{dt}+ (\delta +\beta (\Omega )+\mu )T_3^n =\theta (\Omega ) T_2^n T_3^n, \end{aligned}$$

or

65 $$\begin{aligned} {\frac{dT_3}{dt}}={\theta (\Omega ) T_2 T_3-(\delta +\beta (\Omega )+\mu )T_3}. \end{aligned}$$

Similarly, we can get

66 $$\begin{aligned} {\frac{dT_4}{dt}}={\beta (\Omega ) T_3-\mu T_4} \end{aligned}$$

by applying Taylor’s series on Eq. (38). Hence, we can conclude that our proposed scheme exhibits first-order consistency.

#### Stability of the NSFD scheme

To study the stability analysis, let us assume the following system:

67 $$\begin{aligned} T_1^{n+1}& = C_1=\frac{T_1+h\alpha \mu }{1+h(\gamma +\mu )}, \end{aligned}$$

68 $$\begin{aligned} T_2^{n+1}& = C_2=\frac{T_2+h((1-\alpha )\mu +\gamma T_1)}{1+h(\theta (\Omega )T_3 +\mu )}, \end{aligned}$$

69 $$\begin{aligned} T_3^{n+1}& = C_3=\frac{T_3+h\theta (\Omega ) T_2 T_3}{1+h(\delta +\beta (\Omega )+\mu )}, \end{aligned}$$

70 $$\begin{aligned} T_4^{n+1}& = C_4=\frac{T_4+h\beta (\Omega ) T_3}{1+h\mu }. \end{aligned}$$

The Jacobian matrix corresponding to the system (67)–(70) is

$$\begin{aligned}J=\left[ \begin{array}{cccc} \frac{\partial C_1}{\partial T_1} &{} \frac{\partial C_1}{\partial T_2} &{} \frac{\partial C_1}{\partial T_3} &{} \frac{\partial C_1}{\partial T_4} \\ \frac{\partial C_2}{\partial T_1} &{} \frac{\partial C_2}{\partial T_2} &{} \frac{\partial C_2}{\partial T_3} &{} \frac{\partial C_2}{\partial T_4} \\ \frac{\partial C_3}{\partial T_1} &{} \frac{\partial C_3}{\partial T_2} &{} \frac{\partial C_3}{\partial T_3} &{} \frac{\partial C_3}{\partial T_4} \\ \frac{\partial C_4}{\partial T_1} &{} \frac{\partial C_4}{\partial T_2} &{} \frac{\partial C_4}{\partial T_3} &{} \frac{\partial C_4}{\partial T_4} \\ \end{array} \right] . \end{aligned}$$

Jacobian at the DFE is

$$\begin{aligned}J=\left[ \begin{array}{cccc} \frac{1}{1+h(\gamma +\mu )} &{} 0 &{} 0 &{} 0 \\ \frac{h\gamma }{1+h \mu )} &{} \frac{1 }{1+h \mu )} &{} 0 &{} 0 \\ 0 &{} 0 &{} \frac{1}{1+h(\delta +\beta (\Omega )+\mu )} &{} 0 \\ 0 &{} 0 &{} \frac{h\beta (\Omega )}{1+h\mu } &{} \frac{1}{1+h\mu } \\ \end{array} \right] . \end{aligned}$$

Eigenvalues of the above Jacobian matrix are $\lambda _1=\frac{1}{1+h(\gamma +\mu )}<1$, $\lambda _2=\lambda _4=\frac{1}{1+h\mu }<1$ and $\lambda _3=\frac{1}{1+h(\delta +\beta (\Omega )+\mu )}<1$. Because all eigenvalues are less than one, this validates the desired outcome that the NSFD scheme is stable at the DFE^36^. NSFD schemes do not constitute a singular category of numerical methods. Numerous researchers have expanded upon the Mickens theory. For instance, Gurski^37^ introduced a straightforward mathematical framework for NSFD schemes, specifically tailored for small systems of nonlinear differential equations. This approach leverages conventional techniques used in approximating differential equations, including the incorporation of artificial viscosity and the implementation of a predictor-corrector scheme. In their work, they examined both the NSFD scheme proposed by Mickens and the one developed by Erdogan and Ozis for first-order equations.

## Numerical simulations

**Figure 2:**
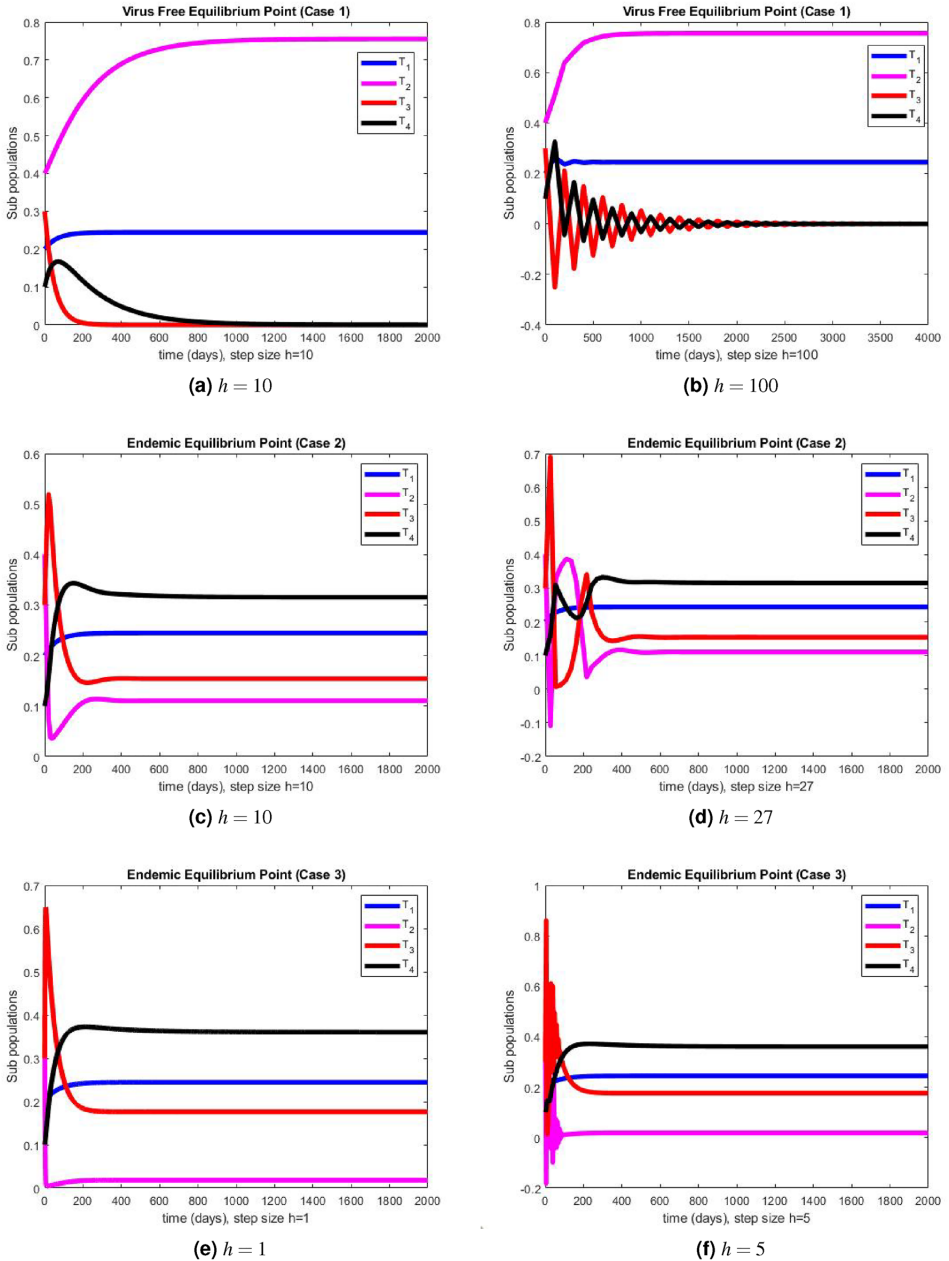
The portions of sub populations using Euler method at different step sizes.

**Figure 3:**
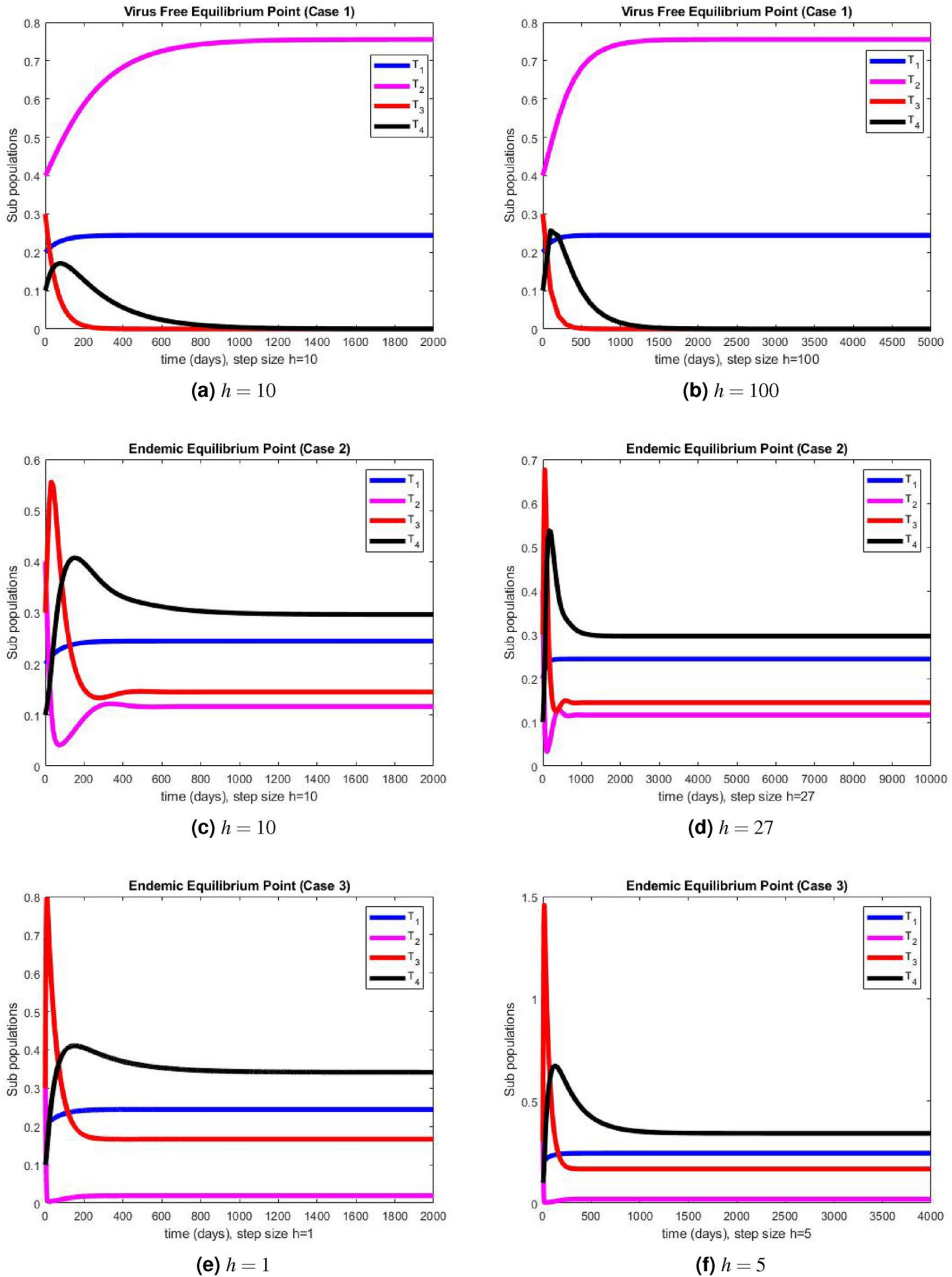
The portions of sub populations using NSFD method at different step sizes.

In this section, we present the simulation results of the performance of Euler method illustrated in Fig. 2a–f, and the simulation results obtained using the NSFD method illustrated in Fig. 3a–f.

The graphical representations of the performance of Euler method are illustrated at various step sizes in Fig. 2a–f. Initially, at a small step size $h=1$, the method exhibits stability, positivity, and convergence. However, as the step size is slightly increased to $h=5$, the method begins to oscillate and generates negative values in all three cases. In models like this, negative values are not meaningful since all compartments represent populations, and negativity is not feasible. Consequently, it can be deduced that this method is not a dependable tool for describing such models. In Fig. 3a–f, we present the simulation results obtained using the NSFD method. This time, the method demonstrates stability, both at smaller and larger step sizes, and an increase in the step size does not adversely affect its convergence and positivity, which are crucial characteristics for modeling disease dynamics. The method consistently displays stable and convergent behavior across all three cases, underscoring its reliability for studying disease dynamics in such conditions. It is worth noting that many classical numerical schemes and their fuzzy counterparts tend to lose their convergence and positivity and struggle to maintain stability as the step size increases. In contrast, the proposed method proves to be more efficient and capable of addressing such issues. We provide the convergence analysis for proposed NSFD scheme analytically and then verify it numerically in numerical simulations section. It can be noted here that the proposed NSFD scheme gives unconditional convergence and remains consistent with the continuous dynamical system. Figure 3a–f show the convergence of NSFD scheme to true equilibria, retaining all the essential features of continuous model unlike the Euler scheme which fails to do so (see Fig. 2a–f).

## Conclusions

In this study, we have considered a typhoid model with fuzzy parameters. We assumed that the infection does not transmit equally among the individual of the populations. Similarly, the treatment rate is also not the same for each individual. As a function of the virus concentrations, we treated the typhoid transmission rate $\theta (\Omega )$ and the treatment rate $\beta (\Omega )$ as fuzzy variables. In deterministic models, these parameters are fixed and independent of the viral load. As a result, it may be said that the fuzzy model is more adaptable and balanced than the crisp system. Fuzzy theory is used to address uncertainty quantification difficulties in mathematical disease modeling. We examined it for various virus loads because fuzzy variables are functions of virus load that depend on virus levels. In light of this, we addressed the studied model’s fuzzy equilibrium points while taking the population’s virus levels into account. We established that the disease-free equilibrium point is reached if the virus concentration is lower than the minimal concentration necessary for disease transmission in the population. When the population’s viral levels exceeded the bare minimum needed for disease transmission, we reached the endemic equilibrium points. For various viral concentrations, the basic reproduction rate is examined. We employed the expected value of a fuzzy number to ascertain the fuzzy basic reproduction number. Two numerical schemes are developed for the approximate solution of the studied model. The developed schemes are analyzed for different amounts of virus. The suggested numerical algorithms must maintain the positive nature of the solutions of such dynamic population models. The Euler method preserved this for only small values of the step sizes and generated negative values by increasing the step size. On the other hand, the NSFD preserved this for all large values of the step size too for different amounts of virus. Additionally, the convergence and consistency of the NSFD scheme are analyzed, demonstrating that the suggested approach is unconditionally convergent and consistent of order 1. The creation, application, and analysis of a non-standard finite difference technique for the numerical analysis of a typhoid illness model with fuzzy parameters are the main foci of the current work. Future developments may include fuzzy stochastic, fuzzy delayed, or fuzzy fractional dynamic senses. Age-structured fuzzy epidemic models could potentially be modeled using the NSFD modeling theory. This research focuses mostly on using triangular fuzzy numbers as membership functions. In the future, we may investigate the use of various fuzzy numbers as potential membership functions, such as trapezoidal, pentagonal, hexagonal, and so on.

## Data Availability

The datasets analyzed during the current study are available from the corresponding author upon reasonable request.
